# Treatment Response in Enteric Fever in an Era of Increasing Antimicrobial Resistance: An Individual Patient Data Analysis of 2092 Participants Enrolled into 4 Randomized, Controlled Trials in Nepal

**DOI:** 10.1093/cid/cix185

**Published:** 2017-02-28

**Authors:** Corinne N. Thompson, Abhilasha Karkey, Sabina Dongol, Amit Arjyal, Marcel Wolbers, Thomas Darton, Jeremy J. Farrar, Guy E. Thwaites, Christiane Dolecek, Buddha Basnyat, Stephen Baker

**Affiliations:** 1 Hospital for Tropical Diseases, Wellcome Trust Major Overseas Programme, Oxford University Clinical Research Unit, Ho Chi Minh City, Vietnam;; 2 Centre for Tropical Medicine and Global Health, University of Oxford, United Kingdom;; 3 Oxford University Clinical Research Unit, Patan Academy of Health Sciences, Lalitpur, and; 4 Global Antibiotic Resistance Partnership, Nepal;; 5 Mahidol Oxford Tropical Medicine Research Unit, Faculty of Tropical Medicine, Mahidol University, Bangkok, Thailand; and; 6 Department of Medicine, University of Cambridge, United Kingdom

**Keywords:** antimicrobial resistance, typhoid, enteric fever, Nepal, fluoroquinolone

## Abstract

**Background.:**

Enteric fever, caused by *Salmonella* Typhi and *Salmonella* Paratyphi A, is the leading cause of bacterial febrile disease in South Asia.

**Methods.:**

Individual data from 2092 patients with enteric fever randomized into 4 trials in Kathmandu, Nepal, were pooled. All trials compared gatifloxacin with 1 of the following comparator drugs: cefixime, chloramphenicol, ofloxacin, or ceftriaxone. Treatment outcomes were evaluated according to antimicrobial if *S*. Typhi/Paratyphi were isolated from blood. We additionally investigated the impact of changing bacterial antimicrobial susceptibility on outcome.

**Results.:**

Overall, 855 (41%) patients had either *S*. Typhi (n = 581, 28%) or *S*. Paratyphi A (n = 274, 13%) cultured from blood. There were 139 (6.6%) treatment failures with 1 death. Except for the last trial with ceftriaxone, the fluoroquinolone gatifloxacin was associated with equivalent or better fever clearance times and lower treatment failure rates in comparison to all other antimicrobials. However, we additionally found that the minimum inhibitory concentrations (MICs) against fluoroquinolones have risen significantly since 2005 and were associated with increasing fever clearance times. Notably, all organisms were susceptible to ceftriaxone throughout the study period (2005–2014), and the MICs against azithromycin declined, confirming the utility of these alternative drugs for enteric fever treatment.

**Conclusion.:**

The World Health Organization and local government health ministries in South Asia still recommend fluoroquinolones for enteric fever. This policy should change based on the evidence provided here. Rapid diagnostics are urgently required given the large numbers of suspected enteric fever patients with a negative culture.

## Summary

This is the largest collection of enteric fever treatment data. The results, from trials conducted in Nepal since 2005, confirm that fluoroquinolones are failing for enteric fever treatment. The World Health Organization enteric fever treatment guidelines should be modified.

Enteric (typhoid) fever is a systemic infection caused by the *Salmonella enterica* serovars Typhi and Paratyphi A, B, and C. Enteric fever is a significant cause of morbidity and mortality in low-income regions [1] and was responsible for an estimated 12.2 million disability-adjusted life-years and >190 000 deaths globally in 2010 [2]. The fatality rate of enteric fever is low (<1%) but is higher when antimicrobial therapy is delayed or unavailable [3]. Therefore, antimicrobials are essential for the clinical management of enteric fever. Chloramphenicol, ampicillin, and cotrimoxazole were first-line treatments for enteric fever until the early 1990s when the increasing incidence of multidrug-resistant (MDR; defined as resistance to these 3 antimicrobial drugs) *S.* Typhi organisms led to the use of fluoroquinolones [4, 5]. Yet, organisms with reduced susceptibility against fluoroquinolones became a problem in Asia soon after their introduction [6, 7]. Recent phylogeographic analyses that document an ongoing epidemic of a global antimicrobial resistance (AMR) *S.* Typhi lineage suggest that the potential for regional or global dispersal of a lineage exhibits resistance to fluoroquinolones is now a real threat [8–10]. In the absence of effective and accessible vaccines and lack of sanitation improvements, development of tailored antimicrobial therapy recommendations is critical to reduce morbidity and prevent disease transmission.

In Kathmandu, Nepal, *S.* Typhi and *S.* Paratyphi A are the most commonly isolated organisms from the blood of febrile adults and children [11, 12]. Over the last decade we conducted 4 randomized, controlled trials (RCTs) to evaluate enteric fever treatment in this endemic region [13–16]. Our aim in this study was to use the largest collection of individual patient data assembled to date from enteric fever treatment trials to evaluate the effect of treatment drug on differences in clinical outcome between *S.* Typhi and *S.* Paratyphi A infections and those with blood culture-negative enteric fever. We further sought to compare the antimicrobial susceptibility profiles over time between *S.* Typhi and *S.* Paratyphi A isolates and to investigate their impact on outcome. An in-depth understanding of trends and clinical implications of AMR enteric fever should guide policymakers and clinicians in decisions regarding treatment in an era of rapidly diminishing therapeutic options.

## MethodS

### Ethical Approval

Written informed consent, which was required for participation in all trials, was provided by a parent or adult guardian if a patient was aged <18 years. The Nepal Health Research Council Ethics Committee and the Oxford Tropical Research Ethics Committee of the United Kingdom provided ethical approval for all 4 studies.

### Patient Populations and Study Procedures

Individual patient data for this study were derived from 4 RCTs conducted at Patan Hospital in Kathmandu, Nepal, between 2005 and 2014, the methods and results of which have been described previously [13–16]. Patients who presented to the outpatient or emergency department with fever lasting longer than 3 days with a clinical diagnosis of enteric fever (undifferentiated fever >38°C with no focus of infection) were eligible. Patients were excluded if they were pregnant or lactating, were aged <2 years or weighed <10 kg, showed any signs of complications (jaundice, shock, gastrointestinal bleeding), showed hypersensitivity to the relevant trial drugs, or had been treated with a study drug in the week prior to going to the hospital. The study procedures between the 4 trials were comparable; however, there were several minor protocol differences between studies (outlined in Supplementary Table 1).

Patients were randomly assigned to 1 of 2 arms in each trial. Each trial was composed of a gatifloxacin arm (10 mg/kg/day, single dose orally for 7 days) and a comparator arm, which was cefixime (20 mg/kg/day, 2 doses orally for 7 days) [13], chloramphenicol (75 mg/kg/day, 4 divided oral doses for 14 days) [14], ofloxacin (20 mg/kg/day, 2 divided oral doses for 7 days) [15], or ceftriaxone (intravenous; 60 mg/kg for patients aged 2–13 years or 2 g for patients aged ≥14 years) [16]. Gatifloxacin was the constant comparator because it is inexpensive and given once daily.

Fever clearance time (FCT) was defined as the time from the first dose of a study drug until the temperature dropped to ≤37.5°C and remained below this temperature for at least 2 days. The composite endpoint treatment failure summarized unfavorable outcomes and was defined as the occurrence of at least 1 of the following: persistent fever (FCT of more than 7 days [trials 1 and 4] or more than 10 days [trials 2 and 3] after treatment initiation), the need for rescue treatment, microbiological failure (blood culture positive for *Salmonella*) on day 8, relapse or disease-related complications within 31 days of treatment initiation, or death. Blood was taken from all patients for microbiological culture on enrollment and on day 8 for culture-positive individuals or those with a potential relapse.

Microbiological investigations have been described previously [13–16]. Blood samples from adult patients were inoculated into media containing tryptone soya broth and sodium polyanethol sulfonate. For children, BacTEC Ped Plus/F bottles were used. Positive bottles were cultured onto MacConkey agar and presumptive *Salmonella* colonies were identified using biochemical tests and serotype-specific antisera. During all 4 trials, minimum inhibitory concentrations (MICs) were determined against the following antimicrobials unless otherwise noted: Augmentin, ampicillin, amoxicillin, azithromycin (2006–2011), cefixime (2005), chloramphenicol, ciprofloxacin (2006–2014), ceftriaxone, gatifloxacin, nalidixic acid, ofloxacin (2006–2014), and cotrimoxazole (2006–2009, 2011–2014), and against tetracycline by E-test (AB Biodisk, Sweden).

### Statistical Analyses

Data from the trials were combined and analyzed using Stata (v 13.1; College Station, Texas). Plots were drawn in R v3.1.1 (R Foundation, Vienna, Austria) using the ggplot2 package. Demographics and clinical variables were tabulated and compared between serovars. Comparisons of clinical parameters between patient populations were structured as logistic regressions with the patient population (either culture positive/negative or *S.* Typhi/*S.* Paratyphi A) as the main covariate and adjustment for age stratum (binary: <16 years/≥16 years). Multivariable models with random effects were fitted to adjust for study heterogeneity as follows: FCT was evaluated using Kaplan-Meier estimates and Cox proportional hazard models with treatment group and age as covariates; logistic regression was used to determine the odds of treatment failure between treatment arms, controlling for age; and linear regression was used to evaluate the relationship between FCT and log_2_ MIC, also controlling for age. Generalized additive models (GAMs; identity link, cubic spline) were used to examine potential nonlinear trends of MIC over time.

## RESULTS

### Baseline Characteristics

**Figure 1.**
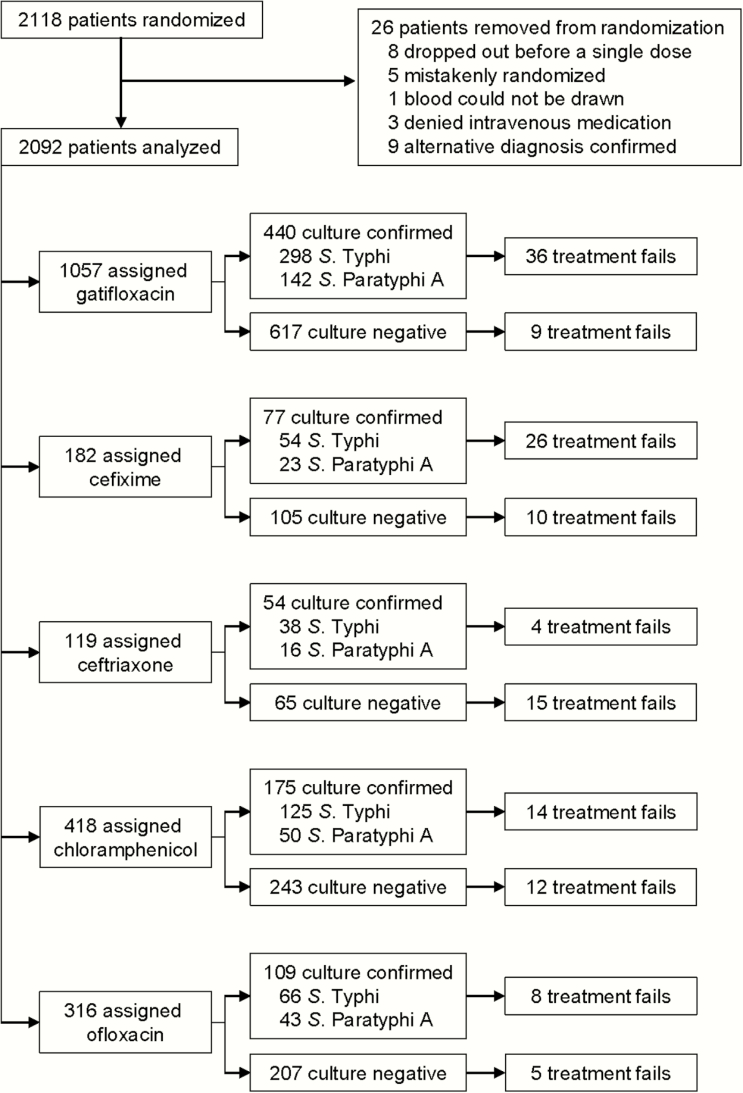
Enrollment of patients into enteric fever treatment trials in Nepal. Flow chart showing enrollment of patients into the 4 individual, randomized, controlled trials according to antimicrobial treatment and blood culture result.

Between 2005 and 2014 there were 2118 patients with clinically suspected enteric fever randomized into 4 trials; data from 2092 (99%) patients were evaluated (Figure 1). Of these, 855 (41%) were culture positive for either *S.* Typhi (n = 581, 28%) or *S.* Paratyphi A (n = 274, 13%). Throughout the study period there were 139 (6.6%) treatment failures including 1 death. The median patient age was 17 years (interquartile range [IQR], 10–23); 66% were male (Table 1). There was no significant difference in age between the culture-negative and culture-positive patients; however, *S.* Typhi patients were significantly younger (median, 16 years; IQR, 9–21) than *S.* Paratyphi A patients (median, 19.5 years; IQR, 13–24) (*P* < .001) (Table 2). There was no difference in the sex distribution between culture-positive/culture-negative and *S.* Typhi/*S.* Paratyphi A populations (Table 2).

**Table 1.** Baseline Characteristics of Patients Enrolled in 4 Enteric Fever Treatment Trials

**Characteristic**	**Trial 1**		**Trial 2**		**Trial 3**		**Trial 4**		**Total**	
	N	n (%) or Median (IQR)	N	n (%) or Median (IQR)	N	n (%) or Median (IQR)	N	n (%) or Median (IQR)	N	n (%) or Median (IQR)
Age (y)	382	17 (9–23)	844	16 (9–22)	623	17 (9–23)	239	19 (15–23)	2088	17 (10–23)
Male sex	382	247 (64.7)	844	540 (64.0)	627	406 (64.8)	239	180 (75.3)	2092	1373 (65.6)
Weight (kg)	382	45 (24–53)	842	42 (21–52)	627	45 (25–54)	237	50 (40–56)	2088	45 (24–53)
Duration of illness before admission (days)	382	5 (3–6)	844	5 (4–7)	625	5 (4–7)	180	5 (4–7)	2031	5 (4–7)
Treatment with antimicrobials in the past 2 weeks	379	238 (62.8)	724	694 (95.9)	623	428 (68.7)	210	109 (51.9)	1936	1469 (75.9)
Previous history of typhoid	382	61 (16.0)	844	138 (16.4)	626	103 (16.5)	238	37 (15.5)	2090	339 (16.2)
Family history of typhoid	382	62 (16.2)	844	140 (16.6)	625	164 (26.2)	239	35 (14.6)	2090	401 (19.2)
Typhoid vaccination	382	2 (0.5)	844	0 (0)	625	0 (0)	238	11 (4.6)	2089	13 (0.6)
Temperature at admission (°C)	379	38.9 (38.3–39.5)	844	38.9 (38.2–39.4)	626	38.6 (38.2–39.0)	235	38.8 (38.3–39.4)	2084	38.8 (38.2–39.4)
Headache	382	370 (96.9)	844	749 (88.7)	627	541 (86.3)	239	211 (88.3)	2092	1871 (89.4)
Anorexia	382	289 (75.7)	844	632 (74.9)	627	455 (72.6)	239	173 (72.4)	2092	1549 (74.0)
Abdominal pain	382	32 (8.4)	844	33 (3.9)	626	25 (4.0)	235	62 (26.4)	2087	152 (7.3)
Cough	382	142 (37.2)	844	277 (32.8)	627	246 (39.2)	239	91 (38.1)	2092	756 (36.1)
Nausea	382	132 (34.6)	844	258 (30.6)	627	174 (27.8)	239	124 (51.9)	2092	688 (32.9)
Vomiting	382	57 (14.9)	844	172 (20.4)	627	118 (18.8)	239	69 (28.9)	2092	416 (19.9)
Diarrhea	382	86 (22.5)	844	161 (19.1)	627	105 (16.7)	239	59 (24.7)	2092	411 (19.6)
Constipation	382	41 (10.7)	844	105 (12.4)	627	79 (12.6)	239	31 (13.0)	2092	256 (12.2)
Hepatomegaly	382	19 (5.0)	844	113 (13.4)	626	7 (1.1)	231	0 (0)	2083	139 (6.7)
Splenomegaly	382	35 (9.2)	844	119 (14.1)	626	6 (1.0)	231	2 (0.9)	2083	162 (7.8)
Haematocrit (%)	370	40 (37–44)	831	39 (36–43)	624	38 (36–42)	235	39 (36–43)	2060	39 (36–43)
Leucocyte count (×10^9^/L)	370	7.0 (5.5–9.0)	831	6.3 (5.0–8.1)	624	6.0 (4.8–7.7)	239	5.9 (4.7–7.3)	2064	6.3 (5.0–8.0)
Platelet count (×10^9^/L)	356	190 (160–235)	800	190 (164–226)	615	174 (145–216)	239	168 (150–209)	2010	184 (153–220)
AST (U/L)	373	47 (36–62)	835	45 (34–61)	624	47 (34–67)	233	49 (36–70)	2065	46 (35–65)
ALT (U/L)	373	33 (24–48)	836	29 (20–43)	624	37 (28–53)	234	45 (31–63)	2067	34 (24–50)
*Salmonella* Typhi isolated	382	119 (31.2)	844	249 (29.5)	627	132 (21.1)	239	81 (33.9)	2092	581 (27.8)
*S.* Paratyphi A isolated	382	50 (13.1)	844	103 (12.2)	627	86 (13.7)	239	35 (14.6)	2092	274 (13.1)
No growth or culture negative	382	213 (55.8)	844	492 (58.3)	627	409 (65.2)	239	123 (51.5)	2092	1237 (59.1)

Trials: 1, gatifloxacin/cefixime [13]; 2, gatifloxacin/chloramphenicol [14]; 3, gatifloxacin/ofloxacin [15]; 4, gatifloxacin/ceftriaxone [16].

Abbreviations: ALT, alanine aminotransaminase; AST, asparate aminotransaminase; IQR, interquartile range.

**Table 2.** Demographic and Clinical Characteristics of Culture-Negative, Culture-Positive, *Salmonella* Typhi, and *S* Paratyphi A Patients

**Characteristic**	**Culture Negative**		**Culture Positive**		*P* Value^a^	***S*. Typhi**		***S*. Paratyphi A**		*P* Value^a^
	N	n (%) or Median (IQR)	N	n (%) or Median (IQR)		N	n (%) or Median (IQR)	N	n (%) or Median (IQR)	
Age (y)^b^	1236	17 (9–24)	852	17 (10–22)	.692	578	16 (9–21)	274	19.5 (13–24)	<.001
Male sex^b^	1237	818 (66.1)	855	555 (64.9)	.565	581	373 (64.2)	274	182 (66.4)	.525
Weight (kg)	1234	44 (23–54)	854	46 (25–53)	.854	580	43.5 (22–52)	274	49 (38–55)	<.001
Duration of illness before admission (days)	1203	5 (4–7)	828	5 (4–7)	.500	565	5 (4–7)	263	5 (4–6)	.102
Treatment with antimicrobials in the past 2 weeks	1146	861 (75.1)	790	608 (77.0)	.330	532	414 (77.8)	258	194 (75.2)	.440
Previous history of typhoid	1236	208 (16.8)	854	131 (15.3)	.276	581	68 (11.7)	273	63 (23.1)	<.001
Family history of typhoid	1236	242 (19.6)	854	159 (18.6)	.657	580	107 (18.4)	274	52 (19.0)	.400
Typhoid vaccination	1234	9 (0.7)	855	4 (0.5)	.511	581	1 (0.2)	274	3 (1.1)	.073
Temperature at admission (°C)	1233	38.7 (38.1–39.2)	851	39 (38.4–39.5)	<.001	577	39 (38.5–39.5)	274	38.8 (38.2–39.2)	<.001
Headache	1237	1098 (88.8)	855	773 (90.4)	.348	581	518 (89.2)	274	255 (93.1)	.237
Anorexia	1237	903 (73.0)	855	646 (75.6)	.190	581	451 (77.6)	274	195 (71.2)	.036
Abdominal pain	1237	479 (38.7)	855	258 (30.2)	.067	581	261 (44.9)	274	97 (35.4)	.061
Cough	1237	495 (40.0)	855	261 (30.5)	<.001	581	193 (33.2)	274	68 (24.8)	.011
Nausea	1237	394 (31.9)	855	294 (34.4)	.310	581	198 (34.1)	274	96 (35.0)	.853
Vomiting	1237	271 (21.9)	855	145 (17.0)	.010	581	106 (18.2)	274	39 (14.2)	.324
Diarrhea	1237	210 (17.0)	855	201 (23.5)	<.001	581	161 (27.7)	274	40 (14.6)	<.001
Constipation	1237	154 (12.4)	855	102 (11.9)	.775	581	63 (10.8)	274	39 (14.2)	.114
Hepatomegaly	1234	84 (6.8)	849	55 (6.5)	.847	578	40 (6.9)	271	15 (5.5)	.804
Splenomegaly	1234	85 (6.9)	849	77 (9.1)	.069	578	48 (8.3)	271	29 (10.7)	.224
Hematocrit (%)	1219	39 (36–43)	841	39 (36–43)	.573	569	39 (35–43)	272	40 (37–44)	.006
Leucocyte count (×109/L)	1220	6.4 (5.0–8.6)	844	6.1 (4.9–7.5)	<.001	572	6.2 (4.9–7.5)	272	5.8 (4.8–7.2)	.528
Platelet count (×109/L)	1187	187 (157–229)	823	180 (150–210)	.002	555	180 (151–214)	268	180 (150–210)	.469
AST (U/L)	1220	42 (32–59)	845	51 (40–69)	<.001	573	54 (42–71)	272	47 (37.5–66)	.023
ALT (U/L)	1220	31 (21–46.5)	847	38 (28–53)	<.001	575	39 (28–53)	272	36 (28–49.5)	.564

Abbreviations: IQR, interquartile range; AST, asparate aminotransaminase; ALT, alanine aminotransaminase.

^a^ *P* values derived from logistic regression (categorical variables) or linear regression (continuous variables) with outcome characteristic of interest and a covariate of culture positivity or serovar, controlling for age (<15 years/≥16 years).

^b^ *P* values derived using Fisher exact test for categorical data and the Kruskal-Wallis test for continuous data (not controlled for age).

There were several significant differences in clinical history between patient populations after controlling for age (Table 2). Culture-negative patients were significantly more likely to report coughing (40%) and vomiting (22%) than culture-positive patients (31% and 17%, respectively). Culture-positive patients, however, reported diarrhea (24%) more often than culture-negative patients (17%) in addition to a higher temperature (median, 39.0°C and 38.7°C, respectively). Among the culture-positive patients, those with an *S.* Typhi infection were significantly more likely to report a history of anorexia (78%), coughing (33%), and diarrhea (28%) compared to the *S.* Paratyphi A patients (71%, 25%, and 15%, respectively) and presented with higher temperatures (median, 39.0°C vs 38.8°C). *Salmonella* Paratyphi A patients were significantly more likely to report a history of previous typhoid illness (23%) compared to *S.* Typhi patients (12%). Additionally, there were several significant differences in hematology parameters between the culture-negative/culture-positive patients and the *S.* Typhi/*S.* Paratyphi A patients (Table 1), despite the majority of the values falling within normal ranges. Asparate aminotransaminase and alanine aminotransaminase were significantly elevated in the culture-positive patients (median, 51 U/L and median, 38 U/L, respectively) compared to culture-negative patients (median, 42 U/L and median, 31 U/L, respectively).

### Treatment Failure

The number of patients who failed treatment in each treatment arm is shown in Table 3. Failure rates between antimicrobial treatment arms were largely similar when stratified by microbiological culture result, with a few notable exceptions. Compared to gatifloxacin, culture-positive patients were significantly more likely to fail treatment when administered cefixime (odds ratio [OR], 10.7; 95% confidence interval [CI], 3.72–30.61; *P* < .001). Culture-negative patients were more likely to fail with cefixime (OR, 7.13; 95% CI, 2.82–18.0; *P* < .001), ceftriaxone (OR, 19.3; 95% CI, 8.02–46.5; *P* < .001), and chloramphenicol (OR, 3.67; 95% CI, 1.52–8.86; *P* = .004) compared to gatifloxacin.

**Table 3.** Proportion of Enteric Fever Patients With Treatment Failure by Culture Result and Treatment

**Treatment Arm**	**Culture Negative**		**Culture Positive**		***Salmonella* Typhi**		***Salmonella* Paratyphi A**	
	Total	n (%)	Total	n (%)	Total	n (%)	Total	n (%)
Gatifloxacin	617	9 (1.5)	440	36 (8.2)	298	26 (8.7)	142	10 (7.0)
Cefixime	105	10 (9.5)	77	26 (33.8)	54	19 (35.2)	23	7 (30.4)
Ceftriaxone	65	15 (23.1)	54	4 (7.4)	38	3 (7.9)	16	1 (6.3)
Chloramphenicol	243	12 (4.9)	175	14 (8.0)	125	11 (8.8)	50	3 (6.0)
Ofloxacin	207	5 (2.4)	109	8 (7.3)	66	7 (10.6)	43	1 (2.3)

### Fever Clearance Times

**Figure 2.**
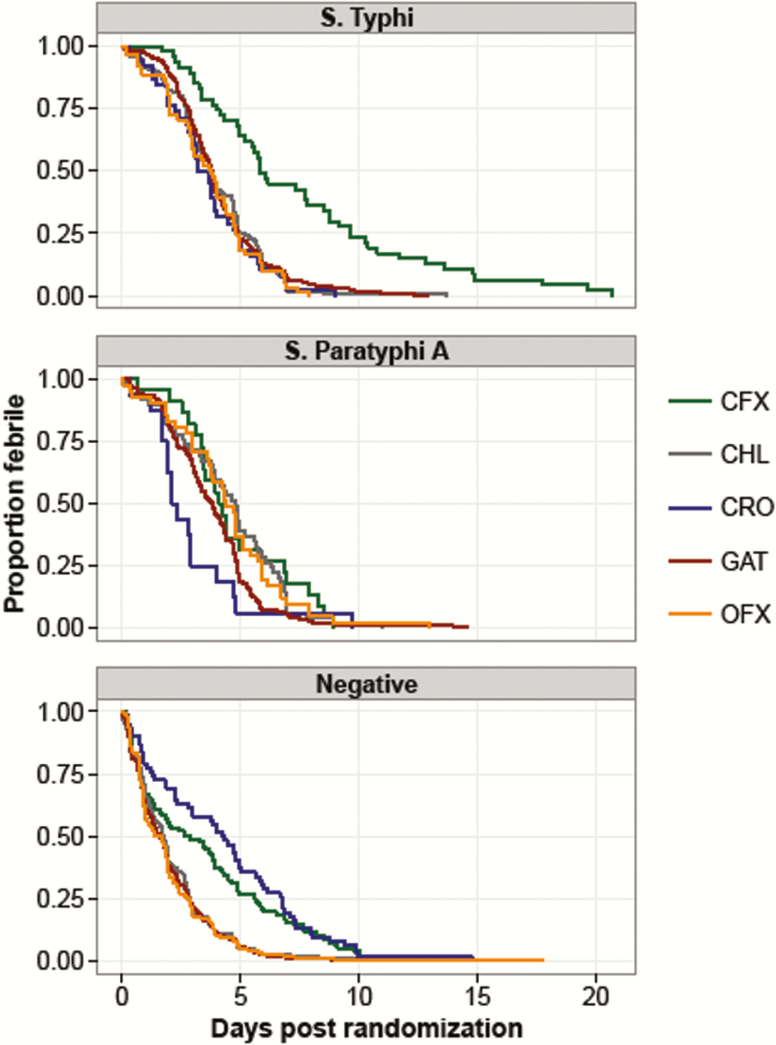
Fever clearance time (FCT) by treatment arm and culture result. FCT (in days) is shown for *Salmonella* Typhi, *S.* Paratyphi A, and culture-negative patients. Colors indicate the different treatment arms. Abbreviations: CFX, cefixime; CHL, chloramphenicol; CRO, ceftriaxone; GAT, gatifloxacin; OFX, ofloxacin.

The FCTs of the various patient populations are shown in Figure 2 and Table 4. Among the culture-positive patient population, *S.* Typhi patients treated with cefixime (hazard ratio [HR], 0.36; 95% CI, 0.25–0.54; *P* < .001) and ceftriaxone (HR, 1.53; 95% CI,1.01–2.31; *P* = .043) had significantly longer FCTs than *S.* Typhi patients treated with gatifloxacin. In the culture-positive patients, those infected with *S.* Typhi also had significantly longer FCTs than *S.* Paratyphi A patients when treated with cefixime (HR, 2.18; 95% CI,1.25–3.80; *P* = .006; Table 4). However, *S.* Paratyphi A–infected patients had longer FCTs when treated with chloramphenicol compared to *S.* Typhi–infected patients (HR, 0.069; 95% CI, 0.49–0.97; *P* = .031). Compared to gatifloxacin, culture-negative patients fared significantly worse when treated with cefixime (HR, 0.56; 95% CI, 0.43–0.71; *P* < .001) and ceftriaxone (HR, 0.42, 95% CI, 0.31–0.57; *P* < .001).

**Table 4.** Fever Clearance Time (in hours) for 4 Enteric Fever Patient Populations by Treatment

**Population**	**Culture Negative**			**Culture Positive**			***Salmonella* Typhi**			***Salmonella* Paratyphi A**		
	N	Median FCT (IQR)	Range	N	Median FCT (IQR)	Range	N	Median FCT (IQR)	Range	N	Median FCT (IQR)	Range
Overall	1178	41.3 (18.2–71.3)	1.0–425.5	810	92.7 (65.3–124.7)	1.0–496.0	549	92.0 (66.4–125)	1.0–496.0	261	94.4 (56.1–122.8)	1.0–349.0
Treatment arm												
GAT	585	39.1 (17.0–68.0)	1.0–285.9	416	90.9 (64.3–116.9)	1.0–349.0	283	90.8 (67.4–117.3)	1.0–309.6	133	91.9 (55.8–116.0)	6.8–349.0
CFX	96	66.5 (18.5–134.5)	4.0–324.0	69	134.0 (82.0–205.0)	16.0–496.0	47	140.0 (96.0–232.0)	40.0–496.0	22	100.0 (81.0–164.0)	16.0–214.0
CRO	62	102.3 (31.5–161.5)	1.0–354.3	54	73.5 (46.0–112.8)	7.8–232.8	38	82.6 (54.0–117.5)	7.8–215.4	16	53.1 (43.3–83.0)	7.8–232.8
CHL	239	41.5 (20.2–68.7)	1.0–304.5	169	94.2 (65.2–136.3)	2.8–327.4	120	89.8 (65.2–121.7)	2.8–327.4	49	114.7 (63.4–151.6)	4.4–262.8
OFX	196	36.8 (17.9–66.4)	1.0–425.5	102	94.8 (56.0–122.3)	1.0–311.8	61	89.8 (48.0–115.4)	3.6–189.8	41	104.4 (71.5–141.6)	1.0–311.8

Abbreviations: CFX, cefixime; CHL, chloramphenicol; CRO, ceftriaxone; FCT, fever clearance time; GAT, gatifloxacin; IQR, interquartile range; OFX, ofloxacin.

### Antimicrobial Susceptibility Trends

**Figure 3.**
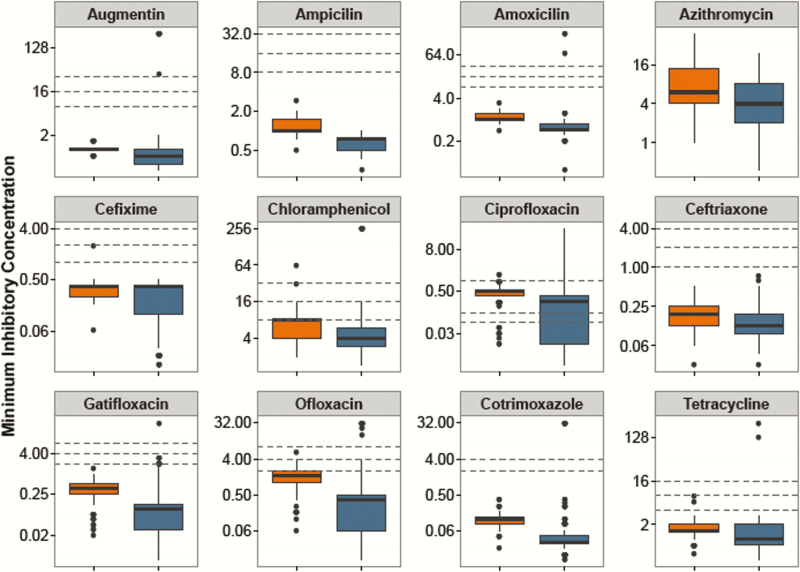
Distribution of minimum inhibitory concentrations (MICs) against antimicrobials for *Salmonella* Typhi and *S*. Paratyphi A. MICs shown on a log_2_ scale against 12 antimicrobials for *S*. Typhi (blue) and *S*. Paratyphi A (orange). Lower, middle, and upper horizontal dashed lines represent the current Clinical and Laboratory Standards Institute cutoffs for susceptible/intermediate and intermediate/resistant, respectively.

**Figure 4.**
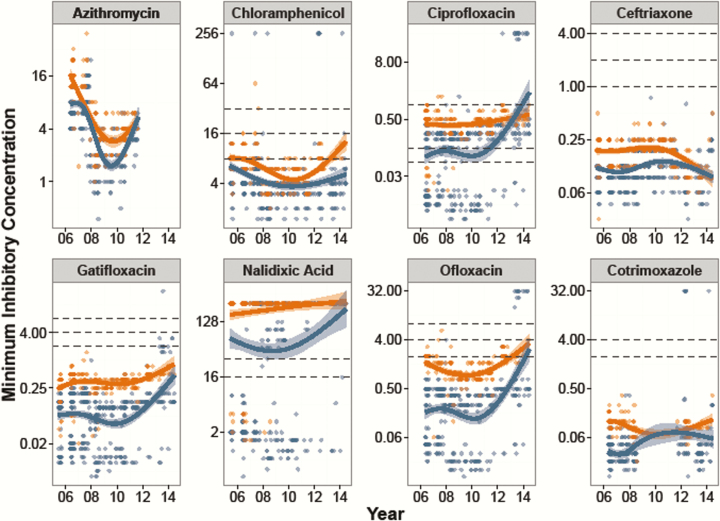
Minimum inhibitory concentrations (MICs) over time for *Salmonella* Typhi and *S.* Paratyphi A. MICs shown on a log_2_ scale for 8 antimicrobials over the period 2005–2014. *Salmonella* Typhi are shown in blue and *S.* Paratyphi A are shown in orange. The smoothed line is derived from the generalized additive model showing a nonlinear increase in MICs over time, with the shaded region showing the 95% confidence interval. Lower, middle, and upper horizontal dashed lines represent the current Clinical and Laboratory Standards Institute cutoffs for susceptible/intermediate and intermediate/resistant, respectively.

As shown in Figure 3, the MICs for *S.* Paratyphi A were significantly higher than those for *S.* Typhi with all antimicrobials (*P* < .001, Kruskal-Wallis), with the exception of cefixime (*P* = .375). Figure 4 shows the MIC time trends by serovar, which were significantly nonlinear over time for all antimicrobials in both serovars (GAM, *P* < .001 with the exception of *S.* Paratyphi A/ciprofloxacin [*P* = .052] and *S.* Paratyphi A/nalidixic acid [*P* = .003]). Most notably, the MICs against the fluoroquinolones rose significantly over time, and the MICs against azithromycin declined between 2007 and 2010. Last, all isolates were susceptible to ceftriaxone throughout the study period.

### Impact of Antimicrobial Resistance on Clinical Outcomes

**Figure 5.**
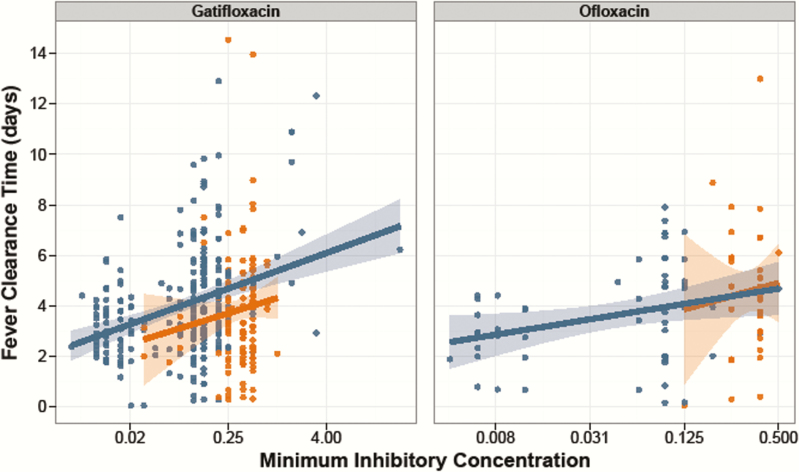
Fever clearance time and minimum inhibitory concentrations (MICs) against fluoroquinolones for *Salmonella* Typhi and *S.* Paratyphi A. Fever clearance time (in days) is shown plotted against log_2_ MIC for gatifloxacin (left) and ofloxacin (right). *Salmonella* Typhi isolates are shown in blue and *S.* Paratyphi A isolates are shown in orange. The lines represent the best-fit linear model, with 95% confidence interval shown by the shaded region.

Increasing MICs against fluoroquinolones led to longer FCTs in *S.* Typhi patients. As shown in Figure 5, an increasing (log_2_) MIC was associated with longer FCTs in patients treated with gatifloxacin (number of hours increase in FCT for each 2-fold increase in MIC (β = 8.1; 95% CI, 5.3–10.8; *P* < .001) and ofloxacin (β = 8.4; 95% CI, 2.2–14.5; *P* = .008). Longer FCTs were also observed with increasing MICs against ciprofloxacin in *S.* Typhi patients treated with ofloxacin or gatifloxacin (β = 6.88; 95% CI, 4.9–8.9; *P* < .001). However, we found no significant association between FCT and (log_2_) MIC against the fluoroquinolones in the *S.* Paratyphi A patients (all *P* > .05). Additionally, there was no significant association between FCT and MIC for the other antimicrobials tested. Last, patients infected with an *S.* Typhi isolate that was nonsusceptible to ciprofloxacin (MIC ≥0.12 μg/mL) were more likely to experience treatment failure (29/211, 13.7%) when treated with ofloxacin or gatifloxacin compared to patients infected with *S.* Typhi organisms susceptible to ciprofloxacin (MIC < 0.12 μg/mL; 2/79, 2.5%; OR, 5.16; 95% CI, 1.1–23.2; *P* = .033). Conversely, we did not identify a similar relationship in those infected with *S.* Paratyphi A (8/149 [5.4%] vs 1/6 [16.7%]; OR, 0.32; 95% CI, 0.03–3.15; *P* = .329), the majority of which exhibited reduced susceptibility against ciprofloxacin (MIC ≥0.12 μg/mL; 211/221, 96%).

## DISCUSSION

Enteric fever remains the leading cause of febrile bacterial illness in Kathmandu [12]. With alarming AMR rates, a lack of immunization as a public health tool, and slow sanitation improvements, tailored antimicrobial therapies for the prevailing AMR profiles are required. Using systematic, longitudinal, individual patient data, we identified dynamic antimicrobial susceptibility profiles among *S.* Typhi and *S.* Paratyphi A isolates and a trend of increasing fluoroquinolone MICs correlating with poor outcome. This phenomenon was particularly apparent among *S.* Typhi patients. Although ceftriaxone was effective in treating culture-confirmed enteric fever patients, we documented poor clinical response in culture-negative patients. These data suggest that careful consideration is required for antimicrobial therapy of patients with enteric fever. In addition, fluoroquinolones should not be recommended for empirical treatment of this infection in South Asia [17].

By combining the largest number of enteric fever patients from a single location, we were able to identify several notable differences in both clinical presentation and clinical response between *S.* Typhi and *S.* Paratyphi A patients. Previous work conducted at the same center showed the 2 serovars to be clinically indistinguishable [18]. We found that, after controlling for age, *S.* Typhi patients were more likely to report anorexia, diarrhea, and coughing and presented with a higher temperature.

The precise mechanism driving the variability in MICs over time for both *S*. Typhi and *S.* Paratyphi A against several antimicrobials throughout 2005–2014 is unknown but may be determined by local prescribing practices. This hypothesis is consistent with notable declines in MDR organisms in both Nepal and India after fluoroquinolones became the first choice of treatment [12, 19, 20]. However, we predict a rapid rebound of MDR organisms with reversion to the prescribing of first-line antimicrobials due to the circulation of MDR plasmids in *S*. Typhi and other organisms [8, 21].

Our study period captured dynamic changes in MICs against fluoroquinolones, particularly among *S.* Typhi isolates in more recent years. Through whole genome sequencing, we have determined that this rise in MIC is associated with the emergence of an H58 variant with mutations in the DNA gyrase gene (*gyrA*) and the DNA topoisomerase IV gene (*parC*) [10, 16]. Supporting these findings, we can conclusively show that FCTs and the rate of treatment failure increases with elevated MICs in *S.* Typhi patients treated with a fluoroquinolone, confirming results from small studies conducted elsewhere [7, 22]. However, although *S.* Paratyphi A isolates had significantly higher MICs against all tested fluoroquinolones in comparison to *S.* Typhi, poor outcome was not significantly associated with increasing MIC. We suggest continued surveillance of *S.* Paratyphi A in the region to monitor for the emergence of high-level fluoroquinolone-resistant organisms similar to trends in the *S*. Typhi population.

As highlighted in our most recent RCT, patients with suspected enteric fever who were blood culture negative were treated effectively with gatifloxacin, yet fared less well when treated with ceftriaxone [16]. The present analysis shows that ofloxacin also performs well in treating those with culture-negative enteric fever. However, due to the low sensitivity of blood culture for the detection of *S.* Typhi and *S.* Paratyphi A[23], it is likely ofloxacin may have been effective against undetected enteric fever cases. We have documented that a reasonable proportion (22%, 21/96) of patients enrolled in the third trial included in the present analysis [14] who were blood culture negative were serologically positive for murine typhus [24]. Doxycycline is considered the drug of choice for rickettsial infections, although it seems that fluoroquinolones may also have clinical activity [24].

In 2003 the World Health Organization published guidelines that recommended azithromycin, ceftriaxone, or cefixime for quinolone-resistant *S.* Typhi and *S.* Paratyphi A infections [23]. Azithromycin is safe and efficacious for the treatment of uncomplicated typhoid [25, 26]. Although there are no current clinical MIC breakpoints, the majority of isolates (88%) here were susceptible, using the previously suggested cutoff of <16 µg/mL [27]. The low MICs against ceftriaxone and rapid FCTs throughout the study period indicate that this drug is likely to be effective for culture-confirmed enteric fever in Nepal. The cost and parenteral route of administration, however, make ceftriaxone less suitable for patient treatment in low- and middle-income countries, particularly as 60%–90% of enteric fever patients are treated as outpatients [3]. An alternative would be the oral, third-generation cephalosporin cefixime. However, our first trial, in which we compared gatifloxacin with cefixime, had to be stopped early by the Data Safety Monitoring Board because of the high failure rate in the cefixime arm (26/77) compared to the gatifloxacin arm (5/92; OR, approximately 9), despite all strains being cefixime susceptible [13]. Our analysis supports a recommendation for azithromycin or ceftriaxone for culture-confirmed enteric fever, and in the absence of rapid diagnostics for rickettsial infections [28], a combination of ceftriaxone and doxycycline in culture-negative febrile patients in this setting [16]. However, identification of extended-spectrum β-lactamase–producing *S.* Paratyphi A in India again suggests vigilance is required.

Our study has limitations. First, the poor diagnostic sensitivity of blood culture may lead to misclassification of a significant number of patients. Although a proportion of culture negatives are likely to be positive for *Rickettsia* spp., this was not directly assessed [24]. Furthermore, by combining patients from individual RCTs with some differing definitions, the data became nonrandomized; however, we included a random effect of study to account for heterogeneity between studies and controlled for age. Therefore, strong associations, such as odds of treatment failure between cefixime and gatifloxacin in culture-positive patients, may be reduced with the larger, nonrandomized data. Additionally, we were unable to access pharmacy records to evaluate the relationship of prescribing patterns for febrile patients and MICs against common antimicrobials. Notwithstanding these limitations, the results from this largest collection of trials with patient recruitment spanning a decade in an endemic location with a high burden of disease will help to inform therapy recommendations.

In conclusion, poor sanitation, low vaccine uptake, and the emergence of extensive ciprofloxacin-resistant *S.* Typhi in Kathmandu suggest that appropriate antimicrobial usage policies are required in order to limit morbidity, mortality, and transmission. In this large evaluation, we document shifting antimicrobial susceptibility profiles, an association between poor treatment outcome, and *S.* Typhi MICs in patients treated with a fluoroquinolone and again highlight the need for better diagnostics for febrile diseases in this setting. We reiterate that fluoroquinolones should not be recommended for the empirical treatment of enteric fever in South Asia [8, 29] and advocate the use of azithromycin or ceftriaxone, in addition to surveillance for changes in AMR profiles.

## Supplementary Data

Supplementary materials are available at *Clinical Infectious Diseases* online. Consisting of data provided by the authors to benefit the reader, the posted materials are not copyedited and are the sole responsibility of the authors, so questions or comments should be addressed to the corresponding author.

## Supplementary Material

Supplementary_Table

## Notes

***Acknowledgments.*** We acknowledge Mangal Rawal, Sumi Munankarmi, Bibek Karki, Radheshyam KC, Sudeep Dhoj Thapa, Rabin Gautam, Priyanka Tiwari, Manisha Risal, Surendra Shrestha, Balmukunda Neupane, Nabraj Regmi, Krishna Prajapati, and Bimal Thapa and the trial monitors Nguyen Thi Phuong Dung and Nguyen Thi Thanh Thuy for their assistance in conducting these trials. We thank Abhusani Bhuju for her painstaking clerical work for this manuscript. We also thank the community medical auxiliaries and all of the patients and their families who participated in the trials.

***Disclaimer.*** The funders had no role in study design, data collection and analysis, decision to publish, or preparation of the manuscript.

***Financial support.*** This project was funded by the Wellcome Trust of Great Britain (106158/Z/14/Z). S. B. is a Sir Henry Dale Fellow, jointly funded by the Wellcome Trust and the Royal Society (100087/Z/12/Z). C. D. was funded by the Li Ka Shing Foundation Global Health Programme at the University of Oxford. A. K. is funded as a leadership fellow through the Oak Foundation.

***Potential conflicts of interest.*** All authors: No reported conflicts. All authors have submitted the ICMJE Form for Disclosure of Potential Conflicts of Interest. Conflicts that the editors consider relevant to the content of the manuscript have been disclosed.
