# Supplementary material for: Treatment Response in Enteric Fever in an Era of Increasing Antimicrobial Resistance: An Individual Patient Data Analysis of 2092 Participants Enrolled into 4 Randomized, Controlled Trials in Nepal
Source: Clin Infect Dis. 2017 Feb 28;64(11):1522–31. doi: 10.1093/cid/cix185 (PMC5434338; doi:10.1093/cid/cix185)
Supplement: Supplementary_Table [file cix185_suppl_Supplementary_Table.docx]

**Supplementary Table 1**: Specific patient population, inclusion criteria, exclusion criteria, study and microbiological procedures for each trial

| **Characteristic** | | **Trial** | | | |
| --- | --- | --- | --- | --- | --- |
|  |  | **1** | **2** | **3** | **4** |
| **Enrolment period** | | Jun 05 - Sep 05 | May 06 - Aug 08 | Jul 08 - Aug 11 | Sep 11 - Jul 14 |
| **Trial** | |  |  |  |  |
|  | Open label | Yes | Yes | Yes | Yes |
|  | 1:1 randomization, no stratification | Yes | Yes | Yes | Yes |
| **Recruiting Hospital** | |  |  |  |  |
|  | Patan | Yes | Yes | Yes | Yes |
|  | Civil Services | No | No | No | Yes |
| **Inclusion criteria** | |  |  |  |  |
|  | Clinically suspected enteric fever: fever for >3 days without focus of infection | Yes | Yes | Yes | Yes |
|  | Age (years) | 2-65 | >2 | >2 | 2-45 |
|  | Weight | - | >10kg | >10kg | - |
|  | Pre-treated with the following as long as they did not show evidence of clinical response | 1) AMP | 1) AMX | 1) AMX | 1) AMX |
|  |  | 2) SXT | 2) SXT | 2) SXT | 2) SXT |
|  |  | 3) CHL |  |  | 3) CHL |
|  | Residence location | 2.5km radius from hospital | 20km^2^ urban Lalitpur | 20km^2^ urban Lalitpur | - |
| **Exclusion criteria** | |  |  |  |  |
|  | Pregnant or lactating | Yes | Yes | Yes | Yes |
|  | Diabetes mellitus | No | No | No | Yes |
|  | Severe infection: obtundation, shock, clinical jaundice, active gastrointestinal bleed | Yes | Yes | Yes | Yes |
|  | History of hypersensitivity to either trial drug | Yes | Yes | Yes | Yes |
|  | Given the following in the previous week | 1) 3rd generation Cephalosporin | 1) 3rd generation Cephalosporin | 1) 3rd generation Cephalosporin | 1) 3rd generation Cephalosporin |
|  |  | 2) Fluoroquinolone | 2) Quinolone | 2) Fluoroquinolone | 2) Fluoroquinolone |
|  |  | 3) Macrolide | 3) Macrolide | 3) Macrolide | 3) Macrolide |
|  |  |  | 4) CHL | 4) CHL |  |
| **Procedures** | |  |  |  |  |
|  | CMA visit twice per day for at least X days or until the patient is asymptomatic | 10 | 10 days in GAT arm, 14 days in CHL arm | 10 | 10 |
|  | Blood draw volume | 5-8mL | 5-8mL | 3mL for <12 years, 7mL for ≥12 years | 3mL for <14 years, 8mL for ≥14 years |
|  | Blood draw timing: day 1 & day 8 if culture positive on day 1 or suggestive relapse | Yes | Yes | Yes | Yes |
|  | Patient attendance at hospital for follow up on | Day 1, 10 &  month 1, 3, 6 | Day 1, 8, 15 &  month 1, 3, 6 | Day 1, 8, 15 &  month 1, 3, 6 | Day 1, 8, 15 &  month 1, 3, 6 |

Trials: 1 – gatifloxacin/cefixime, 2 – gatifloxacin/chloramphenicol, 3 – gatifloxacin/ofloxacin, 4 – gatifloxacin/ceftriaxone; AMP: ampicillin; AMX: amoxicillin; CHL: chloramphenicol; GAT: gatifloxacin; SXT: trimethoprim-sulfamethoxazole; CMA: community medical auxiliary
